# Mood Responses to Passive and Active Motion Leg Cycling Exercise in Healthy Sedentary Young Adults

**DOI:** 10.1155/2020/7282013

**Published:** 2020-02-29

**Authors:** Vernon Bond Jr., Tamrat Retta, Krishna Kumar, James Dorsey, Vasavi R. Gorantla, Richard M. Millis

**Affiliations:** ^1^Department of Health & Human Performance, Howard University, Washington, DC 20059, USA; ^2^Department of Internal Medicine, Howard University, Washington, DC 20059, USA; ^3^Department of Pharmaceutical Sciences, Howard University, Washington, DC 20059, USA; ^4^Department of Neuroscience & Behavioral Sciences, American University of Antigua College of Medicine, Coolidge, Antigua and Barbuda; ^5^Department of Pathophysiology, American University of Antigua College of Medicine, Coolidge, Antigua and Barbuda

## Abstract

Previous studies suggest that passive motion exercise (PME) may be useful for overcoming exercise limitations associated with a sedentary lifestyle, orthopedic disorders, and various other debilitating conditions. Negative mood response is one of the factors that limit a person's ability to exercise. Therefore, this study tests the hypothesis that the mood response associated with PME is not different than the mood response associated with active motion exercise (AME). Eight women and seven men participated in the study and were administrated the Profile of Mood States (POMS) during modes of PME and AME in a randomized order. Outcome of the POMS consisted of the total mood disturbance score [(feelings of tension + depression + fatigue + anger + confusion) − vigor]. ANOVA was used to determine significance of differences in total mood disturbance, oxygen uptake (V.O_2_), and middle cerebral blood flow velocity (MCAv) at baseline and immediately after 30-minute conditions of PME and AME. Postexercise total mood disturbance score was significantly decreased for both conditions (PME baseline 29.2 ± 5.2 vs. postexercise 16.4 ± 6.8, *P* < 0.05) and AME baseline 22.4 ± 4.4 vs. postexercise 13.1 ± 5.2, *P* < 0.05). These senses of changes in feelings were associated with significant physiological increases in V.O_2_ and MCAv during both PME and AME (*P* < 0.05). These results demonstrate that physiological and mood responses to passive and active motion cycling exercise are not different. Future studies should determine whether passive motion cycling exercise is a useful preventive medicine strategy for overcoming various disease-related exercise limitations and counteracting the adverse effects of sedentary lifestyles.

## 1. Introduction

The positive effects of acute physical activity on mood are well established. The available evidence supports the hypothesis that acute exercise can ameliorate a depressed mood, reduce anxiety and enhance cognitive functions, increase perceptions of energy, and decrease those of fatigue [1–5]. A person's ability to perform active exercise is often limited by muscle strength, adiposity, balance, or sarcopenia [6, 7]. There is a growing interest in using motor-driven (passive) motion exercise to overcome the common limitations for active motion exercise. Previous studies have shown that acute 5–30 minute bouts of passive cycling increases energy expenditure to levels consistent with low-intensity walking [8–10]. Acute, short-term passive motion exercise is also reported to increase body oxygen consumption [11] and improve cerebral hemodynamics [12] to levels similar to those associated with active motion exercise. Supine cycling is one such activity wherein the effects of active and passive motion exercise has been compared. Active motion cycling in a supine posture is one entirely initiated by the patient whereas passive supine motion cycling is one in which the subject's legs are moved for them by the cycle, and no effort comes from the subject. An exercise limitation of having a critical illness is shown to be effectively overcome by engaging critically ill patients in passive supine cycling early during their illness. Compared to a standard physical therapy treatment, an exercise regimen of passive supine cycling is reported to result in significantly faster six-minute walk test times in patients who survive critical illness [13]. Similarities in physiological responses between active and passive motion exercise are also documented. Several measures of functional fitness are found to be similarly improved by exercise regimens consisting of 30 min bouts of exercise twice a week for 12 weeks in two groups of well-matched Japanese octogenarians subjected to either active or passive motion exercise [14]. Compared to a randomized control group, a passive range of motion exercise performed 6–8 times in the first 48 hours of hospital admission is shown to improve motor functions measured one and three months after ischemic stroke [15]. Although physiological effectiveness and similarities of passive motion exercise are reported, there are no studies comparing the same active and passive motion exercise for healthy individuals. Such a comparison would be useful for validating passive motion exercise for overcoming the aforementioned and various other exercise limitations and for counteracting the adverse effects of a sedentary lifestyle. Validation studies will, no doubt, need to be performed before passive motion exercise can be accepted and routinely prescribed. The present study is intended to demonstrate the physiological similarities and validate active and passive motion exercise in a healthy population. Based on the aforementioned similarities in oxygen consumption and cerebral hemodynamic improvements reported for active and passive exercise, we hypothesize that active and passive motion exercise may similarly improve a person's mood state. The present study was, therefore, designed to test the hypothesis that passive cycling motion exercise elicits mood responses that are indistinguishable from the mood responses to active motion exercise. We employed thirty-minute bouts of exercise on a motorized leg-cycle ergometer to compare the mood responses of healthy sedentary subjects following passive and active motion exercise.

## 2. Methods

### 2.1. Subjects

Subjects were college students who volunteered following recruitment using an institutionally approved flyer posted throughout the university. Before participation, interested persons completed a medical history form, International Physical Activity Questionnaire (IPAQ) [16], and signed an informed consent form approved by the Howard University Human Participants Institutional Review Board. The medical history and IPAQ were completed during the informed consent process at a time separate from completing a mood questionnaire. For inclusion, participants were required to be healthy young adults with sedentary lifestyle; sedentary defined as not engaging in physical exercise for more than 20 min one day per week. Exclusion criteria included the following: (i) any medication including birth control pills, (ii) body fat > 30%, and (iii) exercise-trained. The subjects consisted of 8 healthy, sedentary young adult women and 7 men aged 21.9 ± 0.2 yrs, height 169 ± 2.5 cm, wt. 81.3 ± 4.4 kg, and body fat 25.1 ± 2.5%. All subjects were of African-American ethnicity.

### 2.2. Study Design

This study was designed as a crossover experiment wherein one group was subjected to conditions of performing a 30 min bout of passive motion exercise (PME) and a 30 min bout of active motion exercise (AME). The participants were randomly assigned, by a coin toss, to the order of performing the two exercise protocols, PME and AME. The randomly assigned bouts of PME and AME were performed at the same time of day separated by one week. The research assistant was not blinded to the test conditions.

### 2.3. Physiological Measurements

Body weight and height were measured using an electronic weight-height scale (SR Scales, SR Instruments, Inc.). Body composition was assessed using dual-energy X-ray absorptiometry (QDR 4500, Hologic, Inc.). Metabolic oxygen consumption (V.O_2_) was measured using an indirect calorimetric method. Respiratory gas concentrations of expired O_2_ and CO_2_ were measured through a mouthpiece connected to an O_2_ and CO_2_ analyzer (Max II; Physio-Dyne Instruments). Minute ventilation and expired gas volumes were measured using a flow turbine (Hans Rudolph, Inc.). The gas analyzers and gas volume turbine were calibrated before each test. End-tidal CO_2_ (ETCO_2_) was measured through a sample line using a CAPNOMAC ll capnometer (Datex Ohmeda, Inc.). Middle cerebral artery blood flow velocity (MCAv) was assessed by transcranial Doppler 6 ultrasonography (EME Pioneer TC 2020; Nicolet Vascular), using a 2 MHz probe placed over the temporal window. The right MCAv signal was identified and tested according to standard criteria guided by signal depth, velocity, and characteristics [17]. The ultrasound probe was fixed at a constant angle and secured with a headband. Physiological measures of MCAv, ETCO_2_, and V.O_2_ were continuously monitored during 30 min of AME and PME, and the values recorded during the last min were used for analysis.

### 2.4. Quantifying Mood Responses

Mood responses were measured before (baseline) and following the bouts of PME and AME; therefore, each participant was assessed by POMS at 3 different time points: baseline, after PME, and after AME. Each participant completed an electronically administered questionnaire quantifying tension, depression, vigor, fatigue, anger, and confusion (Profile of Mood States-Long Form, POMS) [18]. POMS was administered by the BrianMac Sports Coaching website (brianmac.co.uk/poms.htm). Correctness of computations of the total mood disturbance score was ensured by the POMS software.

Subjects were instructed to respond to the POMS “based on how you feel right now.” The total mood disturbance score [(tension + depression + fatigue + anger + confusion) − vigor] was used for analyzing the mood response to both PME and AME. Mood and total mood disturbance scores were inversely related; decreased total mood disturbance score was indicative of improvement in mood.

### 2.5. Passive and Active Motion Exercise Protocols

Participants were instructed to enter the laboratory after a six-hour fast and no physical exercise for 24 h. Further instructions provided to the female participants included testing limited to the luteal phase of the menstrual cycle to limit possibly hormonal confounding effects. Following 10 min of seated rest (baseline), baseline values were recorded for 1 min; then, participants performed one of the two-randomized conditions: passive cycling (PME) or active cycling (AME). Prior to the baseline period, participants were instrumented for measurements of MCAv, V.O_2,_ and ETCO_2_ and then connected to a motorized leg-cycle ergometer (MOTOmed ®). After 10 min rest, baseline measures of MCAv, ETCO_2_, V.O_2_, and POMS were recorded. Immediately following baseline measurements, each subject performed 30 min of completely motor-supported leg cycling (PME) at a pedal rate of 60 rpm or 30 min of voluntary leg cycling (AME) at a pedal rate of 60 revolutions per min (rpm) with the motor disengaged. During AME, the desired pedal rate of 60 rpm was maintained by requiring the subjects to pedal to the audio cues of a metronome. The 30 min bout of cycling was supervised by a research assistant.

### 2.6. Statistical Analyses

Data analyses were performed using the IBM SPSS Statistics® 19.0 (IBM Corporation) software package. We performed a repeated measures ANOVA with Newman-Keuls post hoc testing to compare the baseline values and the differences between baseline and PME and between baseline and AME measurements. Two-tailed *α* level of *P* < 0.05 was the cutoff for significance. Data are presented as means ± standard errors. The average baseline POMS total mood disturbance scores of the present study group, before performance of the PME and AME protocols, were compared to those of a normative study group of college students reported by Nyenhuis and associates [19]. The *P*-value and confidence interval (CI) of the mood disturbance scores difference was computed as the probability of obtaining the difference between two observed means based on the pooled standard deviations of the two independent samples. The *P*-value was computed to be the area of the *t* distribution with *n*_1_ + *n*_2_ − 2 degrees of freedom that falls outside ± *t*. *P* < 0.05 indicated that the two means were significantly different [20]. Baseline-postexercise percent changes were used to determine whether the increments in V.O_2_ and MCAv and the decrement in total mood disturbance scores were different for the two exercise conditions, PME vs. AME (one-tailed *t*-test for means with equal variances). To determine whether the coin toss randomization with crossover experimental design was effective at limiting bias with respect to the mood disturbance scoring, we grouped the participants into subgroups PME and AME according to which of the two exercise conditions was performed first (with the other exercise condition performed one week later). The *t*-test for independent samples and unequal variances was used to determine whether there was a significant difference in the baseline mood disturbance scores of subgroups PME and AME based on which exercise condition was performed first.

## 3. Results

The baseline measurements of body oxygen uptake, cerebral blood flow velocity, end-tidal CO_2,_ and total mood disturbance score made before the bouts of PME and AME were not significantly different (*P* > 0.1, Table 1).

**Figure 1:**
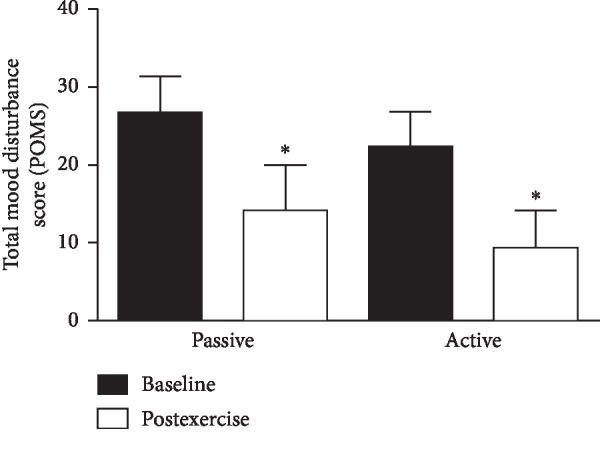
Effects of passive and active motion exercise on mood disturbance score. Bars show the mean ± standard error for total mood disturbance score measured by the Profile of Mood States (POMS) questionnaire. Subjects were 15 healthy sedentary young-adult men and women after control baseline and immediate postexercise periods associated with 30 min bouts of passive and active motion cycling exercise. ^*∗*^Significant at *P* < 0.05.

The total mood disturbance score decreased significantly following PME (postexercise) compared to baseline (29.2 ± 16 vs. 6.4 ± 6.8, *P* < 0.05) and following AME (22.4 ± 4.4 vs. 13.07 ± 5.2, *P* < 0.05) (Figure 1). The total mood disturbance score decrement was not significantly different for PME than for AME (*P* > 0.1).

**Figure 2:**
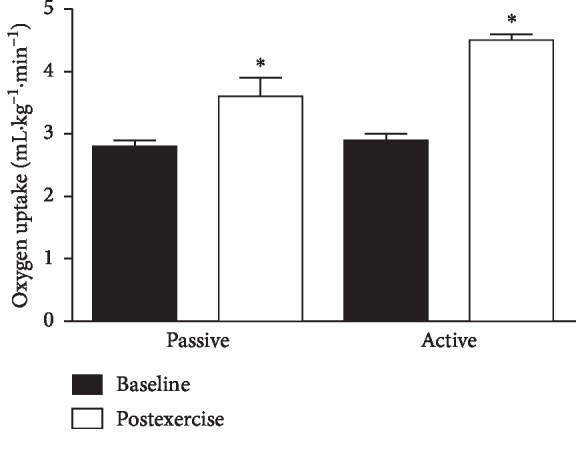
Effects of passive and active motion exercise on body oxygen consumption. Bars show the mean ± standard error for whole body oxygen consumption (V.O_2_). Subjects were 15 healthy sedentary young-adult men and women after control baseline and during the last minute of 30 min bouts of passive and active motion cycling exercise. ^*∗*^Significant at *P* < 0.05.

Body V.O_2_ increased significantly from baseline to the last minute of PME (2.8 ± 0.1 mL·kg^−1^·min^−1^ vs. 3.6 ± 0.3 mL·kg^−1^·min^−1^, *P* < 0.05) and from baseline to the last minute of AME (2.9 ± 0.1 mL kg^−1^·min^−1^ vs. 4.2 ± 0.1 mL kg^−1^·min^−1^, *P* < 0.05) (Figure 2). The AME V.O_2_ tended to be higher compared to PME (4.2 ± 0.1 vs 3.6 ± 0.3 mL kg^−1^·min^−1^), but the difference was not significant (*P* > 0.1).

**Figure 3:**
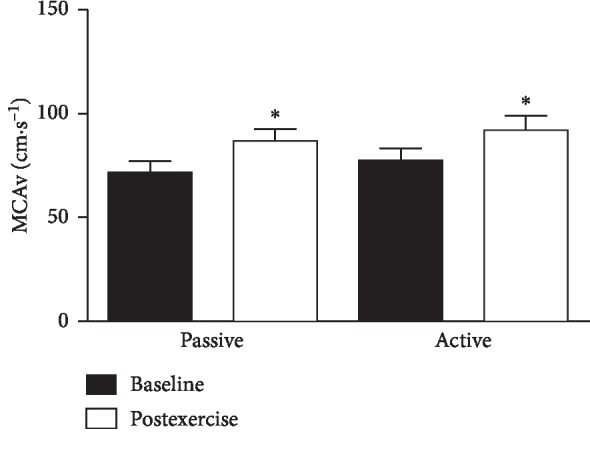
Effects of passive and active motion exercise on cerebral blood flow velocity. Bars show the mean ± standard error for cerebral blood flow velocity measured in the middle cerebral artery (MCAv). Subjects were 15 healthy sedentary young-adult men and women after control baseline and during the last minute of 30 min bouts of passive and active motion cycling exercise. ^*∗*^Significant at *P* < 0.05.

There was a 17.4% increase in MCAv from baseline to the last minute of exercise (*P* < 0.05, Figure 3), and a significant 15.5% increase in MCAv above baseline was found during AME (*P* < 0.05, Figure 3). The increment in MCAv for PME was not significantly different compared to AME (*P* > 0.1).

There were no significant differences in ETCO_2_ between either baseline values or between postexercise PME and AME, with all values ranging 4.0–4.2% (data not shown). That significant changes and condition-related differences in ETCO_2_ values were not found indicates that changes in the MCAv cerebral blood flow measurements were physiological, resulting from the exercise interventions, not from breathing pattern, ventilation, or PCO_2_.

The POMS total mood disturbance scores of the present study group of 28-to-30-year-old college students were not significantly different than those of a normative study group of 18–24-year-old college students reported [19]. The mean baseline mood disturbance scores of the normative study group were 39.9 ± 37.1 (*n* = 132) compared to those of the present study group, 26 ± 19 (*n* = 15). The mean intergroup difference was −13.0 ± 9.72 (SEM) points less for the present study group compared to the normative study group, 95% CI from −33.20 to 5.20 (*P*=0.15, 145 df).

The subgroup that performed the PME first and the AME one week later consisted of 3 females and 2 males; their mean ± SEM baseline mood disturbance score was found to be 24.4 ± 11.2. The subgroup that performed the AME first and the PME one week later consisted of 5 females and 5 males and their mean ± SEM baseline mood disturbance score was 26.1 ± 4.3. There was no significant difference between the baseline mood disturbance scores of the aforementioned subgroups (*P*=0.8).

## 4. Discussion

The rationale for this study was to evaluate the acute effect of passive cycling exercise on mood in healthy participants. We also compared the effects of light-intensity exercise on mood responses of the study subjects. By virtue of the decreased total mood disturbance score observed, our results showed that both types of exercise decreased mood disturbance scores; thereby favorably improving the sense of mood. A coin-toss randomization with crossover experimental design was used. This design appears to have been effective in limiting the potential for bias with respect to the mood disturbance scoring. There was no significant difference between the baseline mood disturbance scores of the participants who performed PME first with AME one week later compared to those who performed AME first with PME one week later.

The present study describes, for the first time, mood-enhancing effects of passive motion exercise in healthy sedentary young adults which appear to be no different than the mood-enhancing effects of active motion exercise. The mood-enhancing effects of passive and active motion exercise are accompanied by increments in oxygen consumption and cerebral blood flow velocity that also appear to be the same as those of active motion exercise. The physiological measurements observed in this study support the hypothesis that passive cycling motion exercise elicits physiological and mood responses that are not different than the mood responses to active motion exercise. These results provide some validation that passive motion exercise may be a useful preventive medicine strategy for overcoming various disease-related exercise limitations and counteracting the adverse effects of sedentary lifestyles.

The comparison of two modes of exercise revealed that both PME and AME significantly increased V.O_2_ above baseline. This finding is similar to that reported previously demonstrating a significant increase in V.O_2_ with passive leg cycling [10, 11]. Compared to baseline measures, an increased cerebral blood flow velocity within the middle cerebral artery (MVAv) was observed during both passive and light-intensity exercise, without a significant difference between the two modes of exercises. There was also no significant difference in end-tidal CO_2_ during the modes of PMS and AMS, thereby indicating that breathing pattern, ventilation, and PCO_2_ did not impact the MCAv measurements significantly. Both middle cerebral artery [21] and regional cerebral blood flow changes, the latter mainly in the anterior cingulate and prefrontal cortices, appear to correlate with changes in cognitive and mood states in healthy humans [22, 23].

To our knowledge, this is the first investigation to show mood improvements based on decrements in POMS mood disturbance scores following both passive and active cycling exercise. It is noteworthy that the POMS total mood disturbance scores for our mixed-gender, 100% African-American nonathlete, sedentary study group of fifteen 28–30-year-old college students is 26 ± 19 (SD). This range is not significantly different (*P*=0.15) than the POMS mood disturbance scores of 40 ± 37 (SD) for a normative group of 132 mixed-gender, 13% African-American, nonathlete 18–24-year-old college-age subjects [19]. We cannot rule out the possibility that this finding represents a trend toward the significant age-related lowering of total mood disturbance scores (improvement in mood) reported [19]. This finding is interesting to us because it raises the question of whether ethnicity-related differences in POMS evaluations of mood states are significant, a hypothesis that has not been systematically studied.

In contrast to previous work that compared light-intensity active cycling to passive cycling, Lindheimer et al. [24] found neither light-intensity exercise nor passive exercise influenced mood. A key difference between this and our studies is that the Lindheimer study explicitly deceived participants about the purpose of their experiment. The mechanisms by which passive cycling exercise might influence mood state are multiple and complex. Various factors are believed to influence the effects of exercise on mood, including participant characteristics, features of the exercise stimulus, and neurobiological and psychological factors [25]. Human studies and rodent models support favorable biological adaptations, including alterations in 5-hydroxytryptamine (5-HT), norepinephrine, galanin, and brain-derived neurotrophic factor, following acute and chronic physical activity [26–28]. Improved mood states have also been reported in response to both acute aerobic and resistance exercise [29, 30]. The magnitude of the improvement in total mood disturbance from baseline following passive cycling exercise (Δ passive exercise = 5% vs. Δ active exercise = 3%) was comparable with light-intensity cycling. The improved mood state following passive leg cycling is likely the result of increased cerebral blood flow, also shown for active leg cycling. Drug-induced increases in cerebral blood flow are reported to produce changes in serotonergic activity [31], consistent with mood enhancement.

### 4.1. Limitations of the Study

The results of this study should be interpreted cautiously because it did not control for placebo effects on mood responses. It is plausible that the mood disturbance scores reported herein are biased by the subjects' knowledge that a goal of the study was their self-reporting of emotional variables. As mentioned previously, one approach to limiting the placebo effect is to intentionally misrepresent the purpose of the study. Indeed, this was cleverly done in at least one study showing no significant changes in either cognitive or mood responses after passive, as well as after active light-intensity cycling [24]. These findings raise the issue as to whether there are alternatives to deception to control for such potential bias. One might think that a nonaerobic type of exercise training might be useful in this regard; however, resistance exercise training is also shown to improve mood responses [32, 33]. These findings seem to suggest that, in the absence of deception, our inability to blind participants to exercise training was unlikely to have avoided producing expectations and psychological perceptions about the active and passive cycling interventions. By the nature of exercise training, we and other researchers must acknowledge that, in the absence of deception, introducing errors to the observed effects of any exercise intervention on mood responses might be unavoidable [34]. Taken together, these findings on placebo effects of exercise training imply that our results are likely to have been biased by perceptions, expectations, and other psychological factors influencing participation of our human subjects. Indeed, any evaluation of mood responses to exercise might be avoided in future studies by adopting the deception approach demonstrated by the study of Lindheimer and associates [24].

## 5. Conclusions

The present study describes, for the first time, a mood-enhancing effect of passive motion exercise which appears to be no different than the mood-enhancing effects of active motion exercise. These mood-enhancing effects are accompanied by equal increments in oxygen consumption and cerebral blood flow velocity which, by virtue of physiological measurements, support the hypothesis that passive cycling motion exercise elicits a mood response that is not different than the mood response to active motion exercise. These results imply that passive motion exercise may be a useful preventive medicine strategy for overcoming various disease-related exercise limitations and counteracting the adverse effects of sedentary lifestyles.

## Figures and Tables

**Table 1:** Baseline values of passive cycling exercise and active cycling exercise.

Variables	Passive cycling exercise	Active cycling exercise
Oxygen uptake (mL·kg^−1^·min^−1^)	2.8 ± 0.4	2.9 ± 0.4
Cerebral blood flow velocity (cm·s^−1^)	71.0 ± 5.6	76.0 ± 6.2
End-tidal CO_2_ (%)	4.3 ± 0.2	4.1 ± 0.8
Total mood disturbance score	29.2 ± 5.2	22.4 ± 4.4

## Data Availability

The raw data represented in this article are available by contacting the corresponding author.
